# Fertility sparing surgery in gestational trophoblastic neoplasia: A report of 4 cases

**Published:** 2016-09

**Authors:** Malihe Hasanzadeh, Ftemeh Vahid Roodsari, Shahnaz Ahmadi, Masoumeh Gavedan Mehr, Tabari Azadeh

**Affiliations:** 1 *Department of Gynecologic Oncology, Women Health Research Center, Ghaem Hospital, Faculty of Medicine, Mashhad University of Medical Sciences, Mashhad, Iran.*; 2 *Department of Obstetrics and Gynecology, Shahid Akbarabadi Hospital, Faculty of Medicine, Iran University of Medical Sciences, Tehran, Iran.*; 3 *Faculty of Medicine, Bushehr University of Medical Sciences, Bushehr, Iran*; 4 *Ghaem Hospital, Faculty of Medicine, Mashhad University of Medical Sciences, Mashhad, Iran.*

**Keywords:** *GTT*, *Fertility*, *Surgery*, *Chemotherapy*

## Abstract

**Background:**

Gestational trophoblastic neoplasia (GTN) is a curable disease that involves the development of malignant tumor in the woman after a normal or molar pregnancy. The position of surgery in GTN is not properly specified and is changing due to new chemotherapy protocols. However, the role of surgery is highlighted in chemotherapy-resistant GTN. Other indications of surgery in trophoblastic diseases are drug toxicity and uterine perforation. Based on the fact that most women in certain age tend to preserve fertility, this study reported 4 cases of successful treatment after fertility sparing surgery.

**Case:**

A hospital-based case-report study was carried out to investigate the role of surgery in 4 patients with GTN. In this study, acute complications, such as intra-abdominal bleeding and liver dysfunction due to chemotherapy occurred in some patients. Surgery was performed and all cases underwent localized tumor removal while preserving the uterus. No hysterectomy surgery was performed.

**Conclusion:**

Surgery is supposed in specific cases of GTN, who desire preserving fertility.

## Introduction

Gestational trophoblastic neoplasia (GTN) most commonly follows a molar pregnancy (1). Myometrial invasion in GTN may be followed by uterine perforation and intraperitoneal bleeding (1). Uterine rupture can be potentially lethal and often entails hysterectomy (2). Surgery in GTN management has been controversial for years, but at present, the role of surgery in GTN treatment is more acceptable. In patients who desire preserving fertility, hysterectomy is usually unacceptable (3). Uterine resection of localized disease with uterine reconstruction can be an alternative, but in certain cases as shown in Table I, primary repair, as a conservative management, should be considered as an option (1). 

There are a few cases of uterine preservation after GTN diagnosis. However, in this study, the most indication of surgery was chemotherapy-resistance GTN or intraperitoneal hemorrhage due to uterine perforation. In patients with uterine rupture or persistent GTN with desire for pregnancy, physicians tend to remove the local uterine lesion due to invasive mole and repair the uterine. Most GTN cases respond to drugs due to new chemotherapy protocols. In rare cases, due to toxicity of chemotherapy drugs, the chemotherapy must be interrupted and the only treatment is surgery.

In this study, four cases of preserving fertility by surgery were considered. Written informed consent was obtained from all patients prior to surgery. 

## Case report

**Case 1**

A 20 year old Iranian woman, NG (nulli gravid) with positive pregnancy test and vaginal bleeding was referred to Ghaem Hospital. Molar pregnancy was revealed by sonography. In 2010/10/23, she was treated by dilatation and curettage, and pathology showed complete hydatiform mole. 

The patient was followed up by weekly β-hCG. Metastatic work up was done as the serum hCG began to rise, and it was found to be negative. The patient had stage 1 GTN and sonography revealed myometrial invasive mole. The patient received two courses of single agent therapy (methotrexate, 50 mg/m^2^ weekly). Due to no response to MTX, the regimen was changed to Actinomycin-D (1.25 mg/m^2^ weekly), followed by five courses of multigene chemotherapy (Etoposide, MTX, Actinomycin-D, Cyclophosphamide and Vincristine), but her serum HCG concentration began to rise again. Transvaginal ultrasonography revealed a 54 mm localized echogen mass with cystic formations that extend to the serous layer of the uterus.

**Figure 1:**
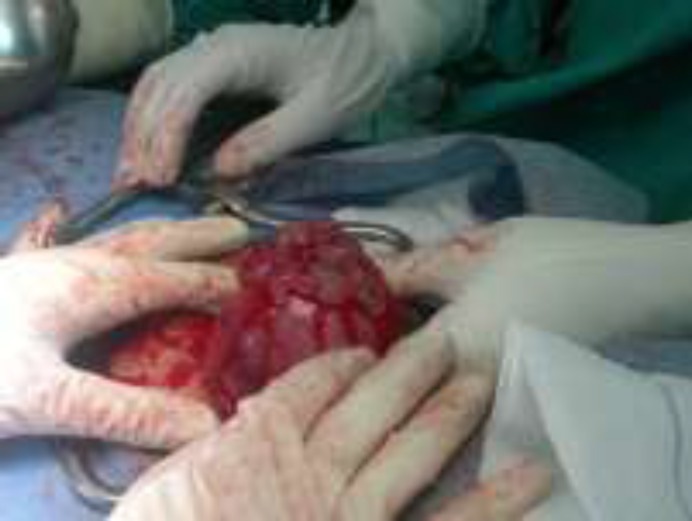
Vesicular mass in fundus of the uterus (480×360

In 2011/3/9, the patient was advised to undergo surgery, since she was very young and had no child and wished to preserve her fertility. Thus she chose to undergo resection of the tumor with uterine reconstruction. An approximately 5×4 vesicular mass was removed from the fundal region of the uterus, thereafter, the uterus was reconstructed in three layers( figure 1,2 ). Her recovery from the surgery was uneventful. Subsequently, the patient received two cycles of EMA-CO( figure 3). The serum HCG concentration rapidly became normal and at present, she is in remission*.*

**Case 2**

Here, we report a suction curettage carried out for a 27 year old Iranian woman (G3Ab2mole1) in 2012/1/2 due to partial hydatiform mole pregnancy. Unfortunately, she was lost to follow up. The patient was in shock (class 3) when she was hospitalized in our center in 2012/4/28. She was noticeably pale with tachycardia and low blood pressure, and pulses usually filiform. The patient immediately underwent exploratory laparotomy and after the evacuation of 3 L of hemoperitoneum and 2 L of clots and vesicular tissues, there was a repair in the left mesosalphynx with active bleeding. There was a 3 cm defect in the fundal area, but the endometrium was intact. Further, the patient was conservatively managed with primary repair of the defect and evacuation of the uterus. A total of 6 units of packed cells and 2 units of FFP were transfused. After surgery, serum βHCG was 20580 mIU/ml. Metastatic work up was done completely. The chest CT scan revealed 3 nodular masses in the right and 2 nodular masses in the left lung. The diagnosis used was GTN stage 3 with a score of six (6). Intramuscularly, chemotherapy was ignited using MTX with a dosage of 50 mg/m^2^ weekly. The patient received 9 cycles of MTX and showed good response to treatment with negative serum β-hCG level. At present, she is in remission.

**Case 3**

A 19 year old Iranian woman with gestational age of 18 weeks was referred to the gynecology oncology department. She had persistent spotting and ultrasound assessment in 2011/3/16, which revealed molar pregnancy. In 2011/3/19, evacuation of the uterus was done by dilatation and curettage in another city. The pathology showed partial hydatiform mole. After the surgery, the patient was not followed-up until 2011/4/4 when her serum βhCG reached 10545 mIU/ml. 

Thus, sonography report cited the probability of pregnancy residual mass and therefore, she was treated with D&C again after one month and the concentration of serum β HCG was found to be higher than 36000 mIU/ml. Metastatic evaluation showed normal chest X-ray and brain CT scan. Ultrasound imaging showed a 30×35 mm mass in the fundus with myometrial involvement, which extended to the serosal layer of the uterus, and a nodular mass in the superior lobe of the left lung was seen in chest CT scan. The diagnosis was GTN stage 1 and single agent chemotherapy was ignited using MTX with a dose of 50 mg/m^2^ weekly.

In 2011/5/10, she underwent an exploratory laparotomy with primary complaint of abdominal pain. The patient was also in shock due to probable uterine rupture. About 1300 ml hemoperitoneal fluid and 300 ml clots were evacuated. The uterus was ruptured in the fundus. There was resection of molar tissue and repair of uterine defect with separated sutures similar to myomectomy repair. The pathology was compatible with invasive mole. She was treated with 11 cycles of MTX and two year after surgery, she had full term pregnancy. Cesarean section was performed and her body was found to be normal and healthy. 

**Case 4**

The fourth case was a 16 year old Iranian woman. About 1 year ago, after a miscarriage, she had continuous spotting and about 3.5 months before being referred to our center, she had been treated by dilation & curettage for molar pregnancy in another country. Thus, during the procedure, the uterus was ruptured and reconstructed by laparotomy. The serum βhCG was increased during her follow up, and her last serum βhCG was 2945 mIU/ml prior to her arrival to our hospital in 2011/10/19. Sonography revealed an echogen mass with the likelihood of a trophoblastic mass. Brain MRI was normal, but the chest CT scan revealed that there were two peripheral nodules of 1.5 and 1.3 cm located in the right lung. Her disease was in stage 3, score 8.Further, the patient was treated with chemotherapy and received three courses of EMA-CO. First, her βhCG decreased, but before the fourth course of chemotherapy, there was a rise in the serum βhCG level, as well as elevated liver enzymes AST: 1664 U/L,ALT;1358 U/Land as a result, her fourth course of chemotherapy was postponed. Eventually, due to persistent elevated liver enzymes, she underwent surgery in 2012/3/3. During laparotomy, some necrotic tissues and a mass were seen on the posterior surface of the uterus (Figure 4A, 4B). The necrotic tissues and then the mass were resected completely and the uterus was reconstructed. In the follow up, her serum βhCG was reached to zero after 2 weeks and as such, did not receive post operation chemotherapy. The serum βhCG in further follow up was normal. 

**Table I:** Review of GTN cases with fertility sparing surgery

**Publication**	**Case**	**Mode of surgery**	**Chemotherapy after surgery**	**Etiology of surgery**
Behtash *et al* (5)	18yr-G1P0	Uterus localized resection	No chemotherapy	Acute abdomen & shock
	19yr-G1P0			
Rojas-Espaillat *et al* (7)	18yr-G1	Lesion removal	2 courses of multi agent chemotherapy	Chemo resistant GTN
Estrella et al (3)	-23yr-G1P0	Mass resection & Uterine repair	MTX change to Actinomycin -D (5 cycles)	Tumor rupture & hemoperitoneum in Ultra sonography
	-23yr-G1P0		MTX 0.4 mg/m^2^ for 5 days (9 cycles)	
Tjalma *et al* (6)	16yr-G1P0	Localized resection	3 cycles of EMACO	Chemo resistant choriocarcinoma
Case *et al* (8)	30yr-G5P3	Localized resection	No chemotherapy	Persistent choriocarcinoma
Current issue	-20yr-G1P0	Localized mass resection	2 cycles of EMACO	Chemo resistant GTN
	-27yr-G3AB2P0	Molar tissue resection	9 cycles of MTX(50mg/m2 weekly)	Shock (class 3)
	-19yr-G1P0	Uterine defect primary repair	11 cycles of MTX	abdominal pain & shock under chemotherapy
	-16yr-G1P0	Mass Resection	No chemotherapy	persistent liver toxicity by chemotherapy

**Figure 2:**
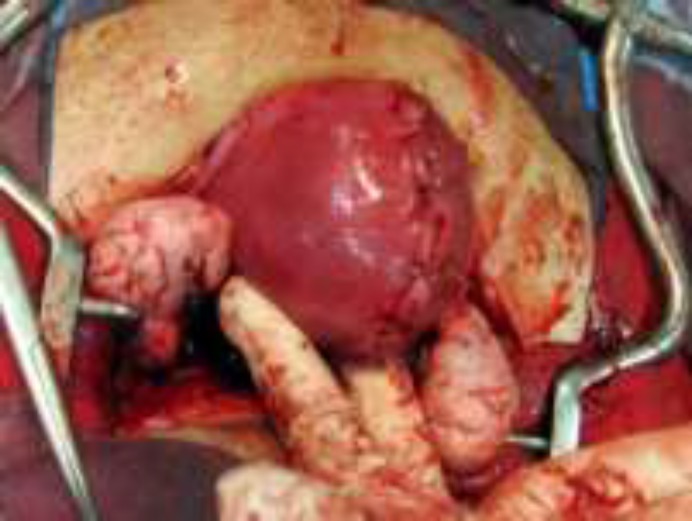
After reconstruction of the uterus (1500×1125

**Figure 3 (A, B).**
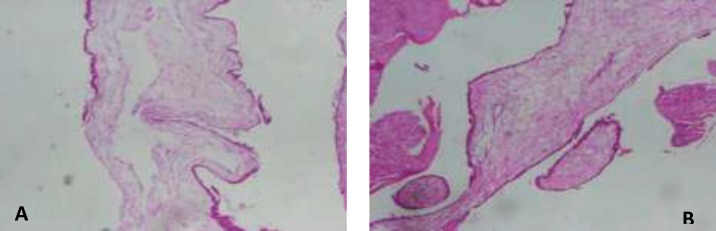
Pathology showed complete mole demonstrating enlarged villous with central cavitation and surrounding trophoblastic hyperplasia. (1500×1125

**Figure 4 (A, B).**
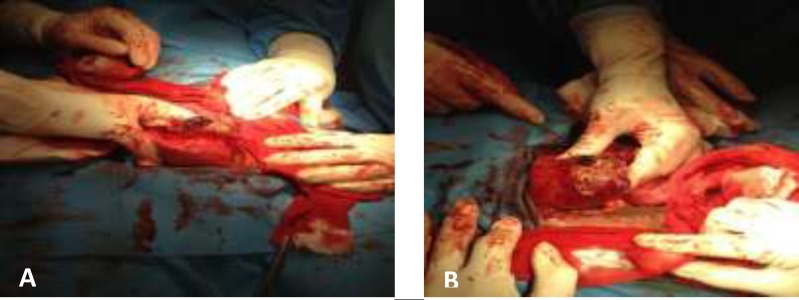
Mass with necrotic tissues surface of the uterus (1009×1079)

## Discussion

All the aforementioned cases were young individuals who desired to preserve their uterus and fertility. The patients were offered the chance to preserve fertility with close follow up. Fortunately, they had complete response after chemotherapy and surgery and therefore, hysterectomy was not required. In general, there was 100% survival of patients. In GTN urgent surgery due to uterine perforation, most physicians perform hysterectomy, but the results of this study and other case report suggested that hystorotomy and mass resection should be considered in young patients (3). The surgeon must be an expert in this field because during resection, much part of the uterus should be left for future fertility (4). 

Few studies have been carried out in which the localized uterine resection of a residual disease was reported, followed by uterine reconstruction. The patient involved young women who desire fertility preservation. Behtash *et al* presented two cases of young patients (18 and 19 years old) with acute abdominal pain and shock, while they were under chemotherapy due to persistent trophoblastic disease. In the emergent exploratory laparotomy, localized resection of the uterus was performed. They had their first successful pregnancy 5 and 4 years, respectively, after surgery (5). However, in this study, one of our cases had successful pregnancy after repair of the uterus. The myometrium may be perforated due to trophoblastic tumor, causing intraperitoneal hemorrhage. GTN with uterine rupture could be potentially lethal and often entails hysterectomy. This treatment, however, may be unacceptable to a woman who wishes to maintain her fertility (3). 

Estrella *et al* reported two cases of uterine rupture in low risk GTN, which was conservatively managed with repair of primary uterine rupture and postoperative single agent chemotherapy. Both patients were in their early reproductive years with a great desire to preserve future fertility (3). In this study, two of cases had uterine perforation and intraperitoneal hemorrhage. The sonographic characteristic in invasive mole and placental site trophoblastic tumor is the same and as such, it is mandatory to have histologic diagnosis before performing definitive surgery (Table I) (6).

## Conclusion

Surgery should be considered in some cases: persistent GTN and no response to chemotherapy, multiple chemotherapy course, and severe toxicity due to chemotherapy. 

## Conflict of interest

The author declares that he has no competing interests.
